# Supplementary material for: Phenylethylamides derived from bacterial secondary metabolites specifically inhibit an insect serotonin receptor
Source: Sci Rep. 2019 Dec 30;9:20358. doi: 10.1038/s41598-019-56892-z (PMC6935581; doi:10.1038/s41598-019-56892-z)
Supplement: Supplementary file 1 — Supplementary Information [file 41598_2019_56892_MOESM1_ESM.pdf]

# Supplementary data

# Phenylethylamides derived from bacterial secondary metabolites specifically inhibit an insect serotonin receptor

**Ariful Hasan[1], Hyun-Suk Yeom[2], Jaewook Ryu[2], Helge B. Bode[3] & Yonggyun Kim[1,*]**

[1]Department of Plant Medicals, College of Life Sciences, Andong National University, Andong 36729, Korea. [2]Center for Eco-Friendly New Materials, Korea Research Institute of Chemicals Technology, Yuseong, Daejeon 34114, Korea. [3]Department of Biosciences, Molecular Biotechnology, and Buchmann Institute for Molecular Life Sciences (BMLS), Goethe-Universität Frankfurt am Main, Germany

* Corresponding author: hosanna@anu.ac.kr

**Supplementary Fig. 1** Putative domain and motif structures of Se-5HTR. Putative seven transmembrane domains (TM1-TM7) are primarily marked by dark yellow bars and circles. Extracellular and cytosolic regions are denoted by green and light blue regions, respectively. Potential N-glycosylation sites and phosphorylation sites are marked by orange and red circles, respectively. Aspartic acid residue (dark blue circle) in TM3 and serine residue (light brown circle) in TM5 are putative residues that might chemically interact with 5-HT. The unique consensus sequence motif (PXXXWXPXF, dark brown circles) in aminergic receptors is conserved in TM6. The motif (NPXXY, dark blue circles) is conserved in TM7 like other GPCRs. Two possible post-translational palmitoylation cysteine residues (light yellow circles) and a PDZ-domain binding motif (ESFL, black circles) are also present in the C-terminal. Conserved motifs were determined using InterPro tool (https://www.ebi.ac.uk/interpro/) and Prosite (http://prosite.expasy.org/) whereas other residues and motifs were predicted using several tools from DTU bioinformatics.

**Supplementary Fig. 2** Potent screened chemicals from HB compounds with their chemical structures and respective median inhibitory concentrations ($IC_{50}$). HB chemicals were injected in different doses (0, 0.01, 0.1, 1, and 10 µg/larva) along with a fixed 5-HT concentration (1 µg/larva) and FITC-tagged bacteria (500 cells/larva). After 15 min of treatment, hemocytes from treated larvae were collected in ACB followed by phagocytosis assay as described above. Percentages of phagocytosis against HB chemical treatment with increasing concentrations were calculated. Their $IC_{50}$ values were determined using Probit analysis ( https://probitanalysis.wordpress.com ).

**Supplementary Fig. 3** Designing a potent chemical inhibitor from phenylehtylamide (PEA) derivatives. Derivatives of PEA were tested for their nodulation inhibition percentages and sorted

by their X and Y groups. Most potent residues inhibiting nodulation were selected and a hypothetical most potent PEA chemical was designed. (A) Core PEA structure with two variable hypothetical residues (X and Y). X belongs to the residue group attached with para position of the phenyl ring whereas Y belongs to the residue group linked to the amide group. (B) Comparative analysis between X residue groups with their mean percent inhibition of nodulation. (C) Comparative analysis between Y residue groups with their percent inhibition of nodulation. (D) A hypothetical PEA chemical compound structure having the most potent inhibition capability.

**Supplementary Fig. 4** Bacterial secondary metabolites (37 chemicals) derived from *Xenorhabdus* and *Photorhabdus*

**Supplementary Fig. 5** Phenylethylamide (PEA) derivatives (45 chemicals) based on HB 44, a bacterial metabolite

**Supplementary Fig. 6** Chemical synthesis of PhX ((*S*)-2-(1,4-dioxohexahydropyrrolo[1,2-*a*]pyrazin-2(*1H*)-yl)-*N*-(4-methoxyphenethyl)acetamide)

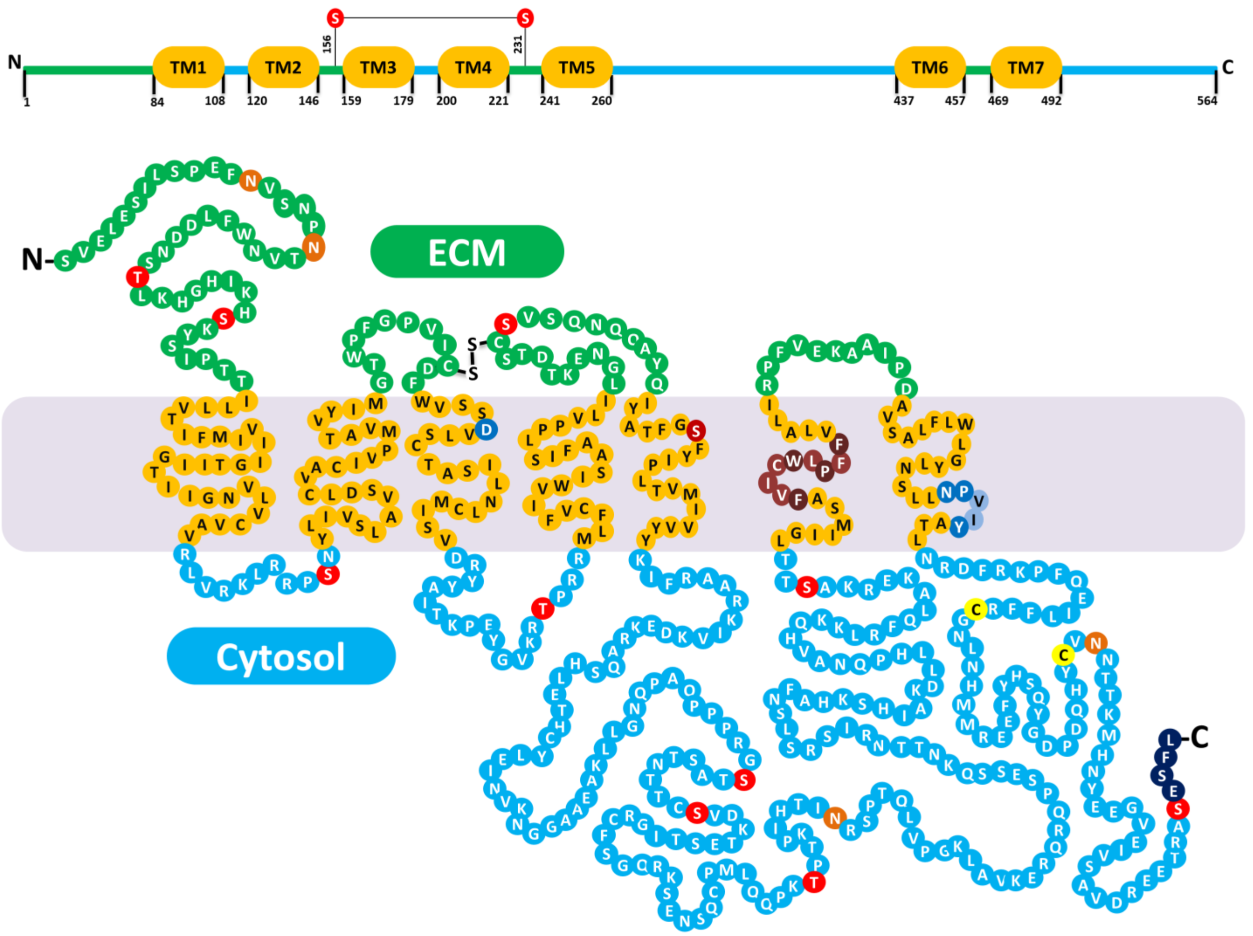


**Supplementary Fig. 1**

| Groups | ID | Structure | $IC_{50}$ (μM) |
|---|---|---|---|
| Phenylethylamide | HB 4 | 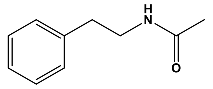 | 253.2 ± 38.0 |
| | HB 5 | 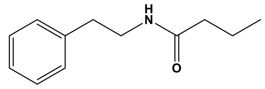 | 217.8 ± 43.6 |
| | HB 44 | 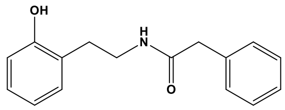 | 103.7 ± 18.7 |
| Tryptamide | HB 23 | 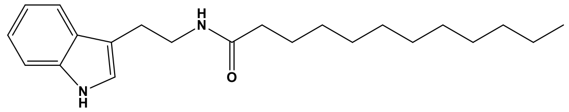 | 103.9 ± 15.6 |
| | HB 50 | 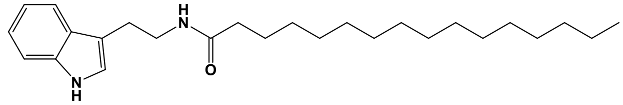 | 90.6 ± 16.3 |
| | HB 531 | 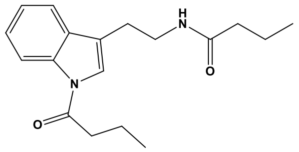 | 124.9 ± 23.7 |
| Xenortide | HB 30 | 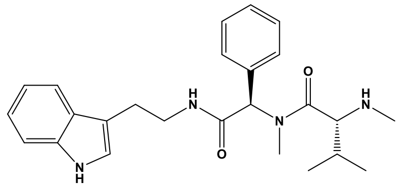 | 59.1 ± 9.5 |
| Xenocyloin | HB 45 | 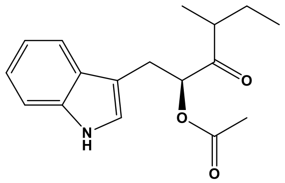 | 82.1 ± 14.8 |
| Nematophin | HB 223 | 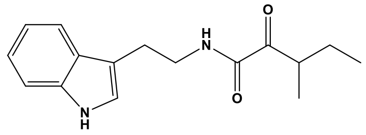 | 134. 6 ± 22.9 |
| GameXPeptide | HB 302 | 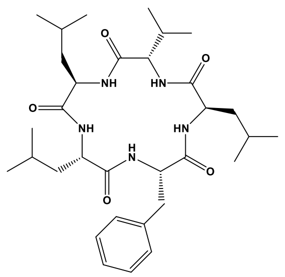 | 58.4 ± 11.7 |

**Supplementary Fig. 2**

**(A)**

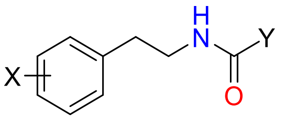


**(B)**

| X | N | Inhibition of nodulation |
|---|---|---|
| 4-$OCH_3$ | 2 | 80.9% |
| 3,4-di-$OCH_3$ | 1 | 53.4% |
| 4-Cl | 3 | 49.6% |
| 4-F | 1 | 72.1% |
| 4-$CF_3$ | 1 | 66.9% |
| 4-H | 2 | 62.7% |

**(C)**

| Y | Inhibition of nodulation |
|---|---|
| (X=4-Cl) | |
| 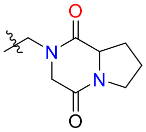 | 84.1% |
| 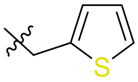 | 36.3% |
| 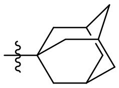 | 35.1% |
| (X=3,4-di-OMe) | |
| 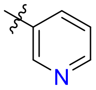 | 77.7% |
| 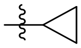 | 77.3% |
| 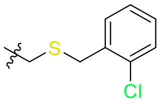 | 53.4% |
| (X=4-F) | |
| 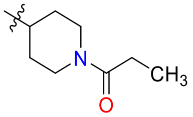 | 72.1% |
| (X=4-$CF_3$) | |
| 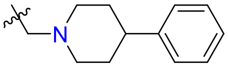 | 66.9% |
| (X=4-H) | |
| 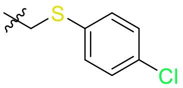 | 64.1% |
| 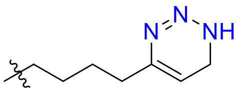 | 61.4% |

**(D)**

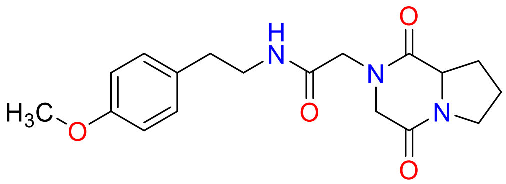


**Supplementary Fig. 3**

| HB Compound | Structure | HB Compound | Structure |
|---|---|---|---|
| HB 4 | 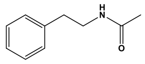 | HB 250 | 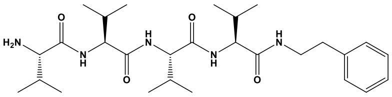 |
| HB 5 | 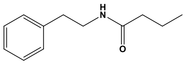 | HB 251 | 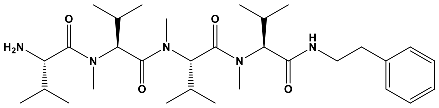 |
| HB 23 | 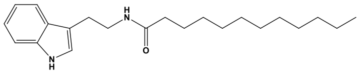 | HB 254 | 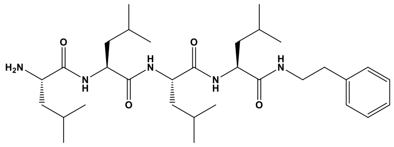 |
| HB 30 | 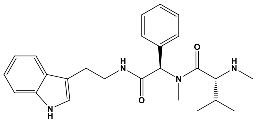 | HB 255 | 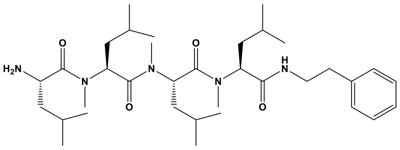 |
| HB 44 | 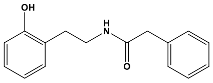 | HB 261 | 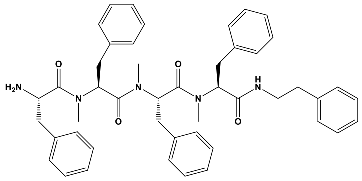 |
| HB 45 | 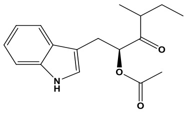 | HB 272 | 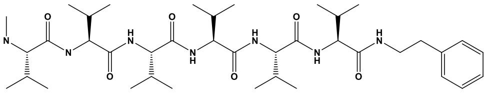 |
| HB 50 | 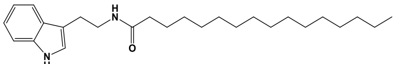 | HB 280 | 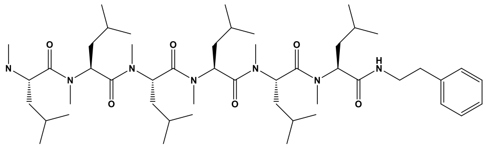 |
| HB 51 | 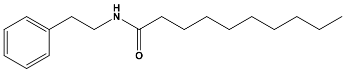 | HB 302 | 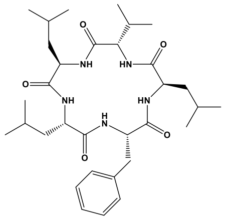 |
| HB 52 | 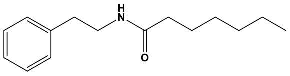 | HB 314 | 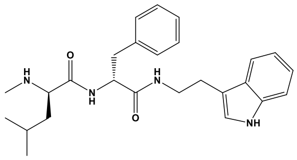 |
| HB 55 | 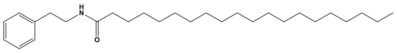 | HB 343 | 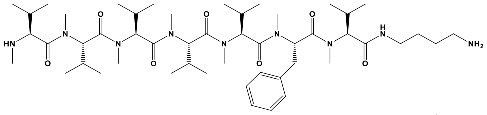 |
| HB 56 | 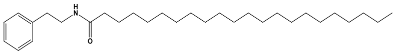 | HB 351 | 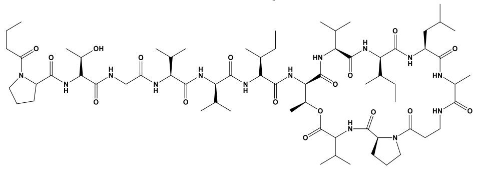 |

HB 58

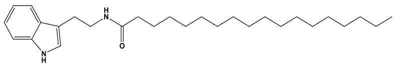

HB 62

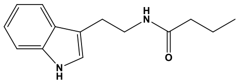

HB 64

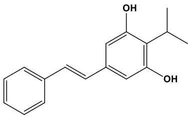

HB 167

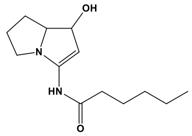

HB 169

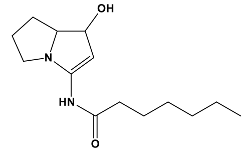

HB 170

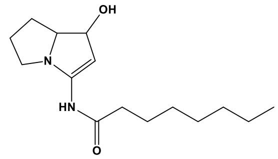

HB 171

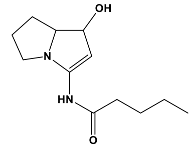

HB 223

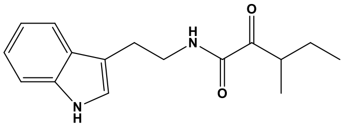

HB 352

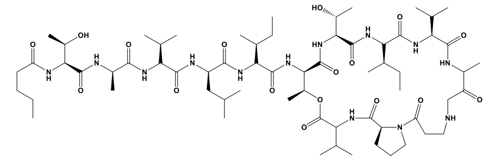

HB 354

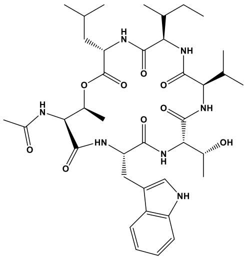

HB 372

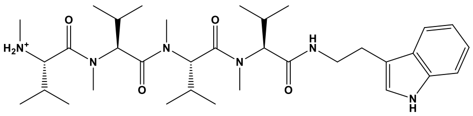

HB 378

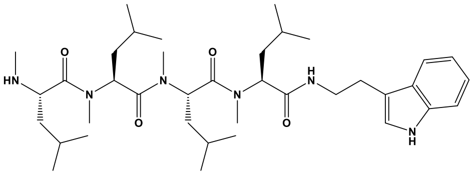

HB 528

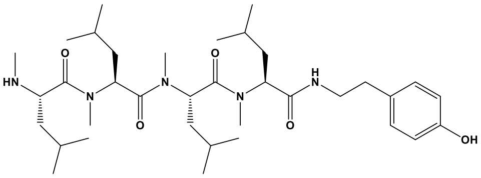

HB 531

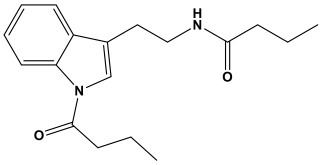

HB 602

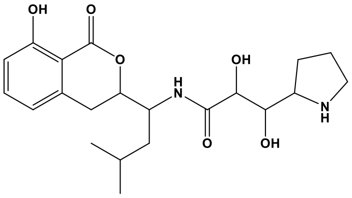

**Supplementary Fig. 4**

| PEA compound | Structure | PEA compound | Structure |
|---|---|---|---|
| Ph 1 | 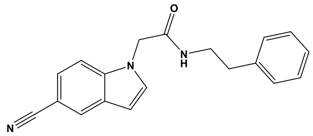 | Ph 24 | 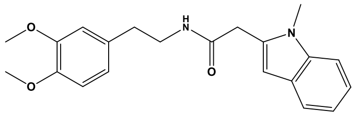 |
| Ph 2 | 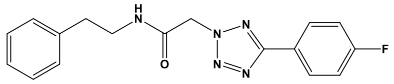 | Ph 25 | 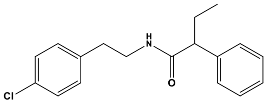 |
| Ph 3 | 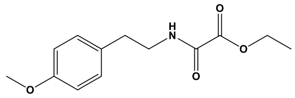 | Ph 26 | 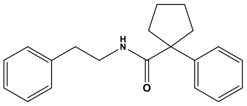 |
| Ph 4 | 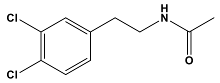 | Ph 27 | 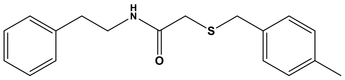 |
| Ph 5 | 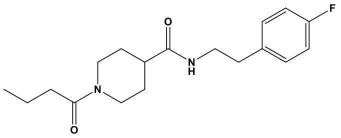 | Ph 28 | 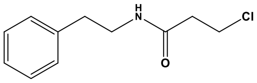 |
| Ph 6 | 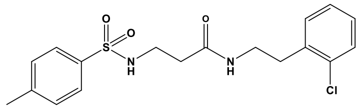 | Ph 29 | 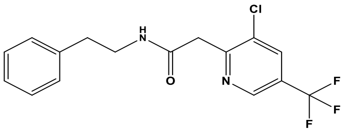 |
| Ph 7 | 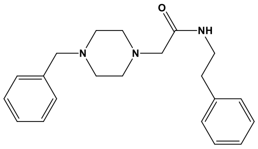 | Ph 30 | 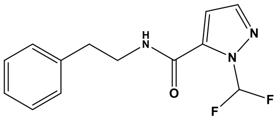 |
| Ph 8 | 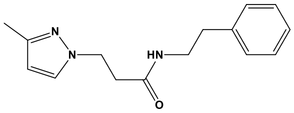 | Ph 31 | 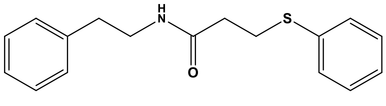 |
| Ph 9 | 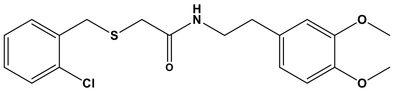 | Ph 32 | 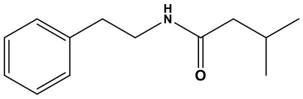 |
| Ph 10 | 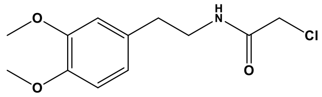 | Ph 33 | 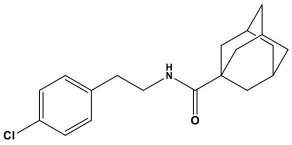 |
| Ph 11 | 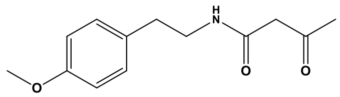 | Ph 34 | 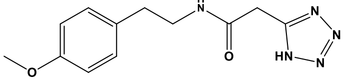 |
| Ph 12 | 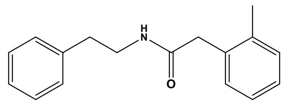 | Ph 35 | 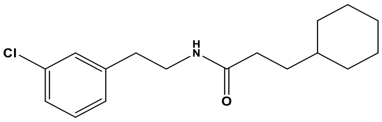 |
| Ph 13 | 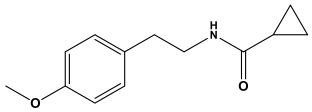 | Ph 36 | 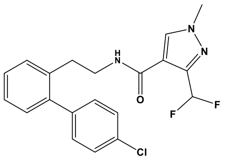 |

Ph 14

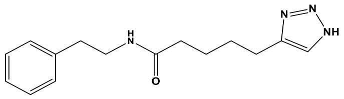

Ph 15

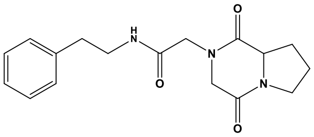

Ph 16

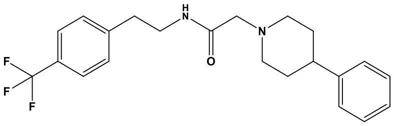

Ph 17

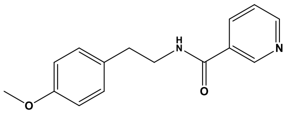

Ph 18

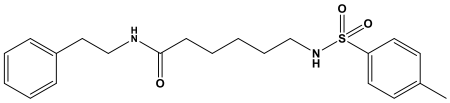

Ph 19

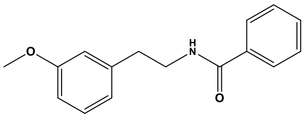

Ph 20

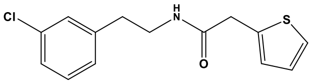

Ph 21

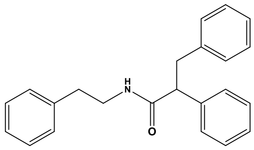

Ph 22

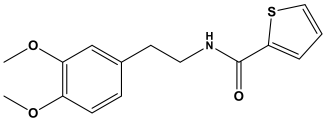

Ph 23

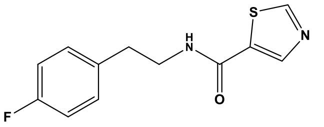

Ph 37

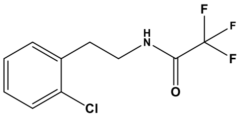

Ph 38

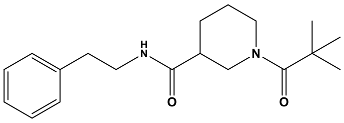

Ph 39

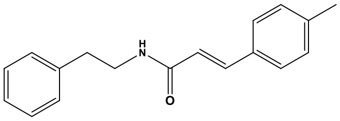

Ph 40

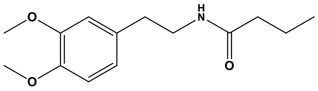

Ph 41

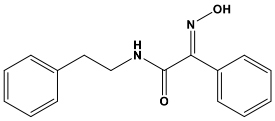

Ph 42

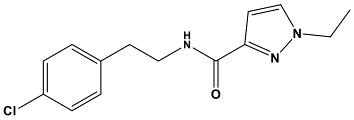

Ph 43

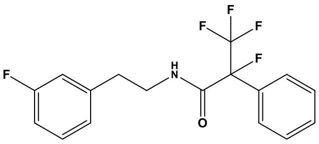

Ph 44

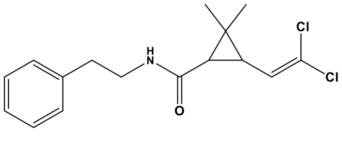

Ph 45

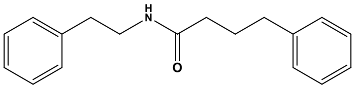

**Supplementary Fig. 5**

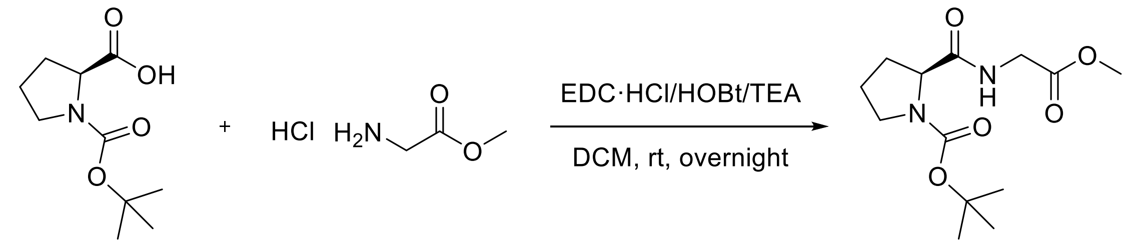


**1** **2** **3**

**Step 1**

Synthesis of (*S*)-(-)-*N*-(*t*-butoxycarbonyl)-prolylglycine methylester (**3**)

To a solution of (S)-(-)-N-(t-butoxycarbonyl)-proline **1** (5.00 g, 23.0 mmol) in $CH_2Cl_2$ (150 mL), amino acid methyl ester hydrochloride **2** (2.89 g, 23.0 mmol), 1-[3-(dimethylamino)propyl)]-3-ethylcarbodiimide hydrochloride (5.34 g, 28.0 mmol), and 1-hydroxybenzotriazole monohydrate (3.77 g, 28.0 mmol) triethylamine (6.50 mL, 46.6 mmol) were added and the reaction mixture was stirred for 24 h at room temperature. The solvent was removed under reduced pressure. The residue was taken up in ethyl acetate (200 mL) and washed successively with water (200 mL), 1N HCl aqueous solution (200 mL), and sat. $NaHCO_3$ aqueous solution (200 mL). The solvent was removed under reduced pressure to give (*S*)-(-)-*N*-(*t*-butoxycarbonyl)-prolylglycine methylester **3** as a white solid (4.82 g, 73%).

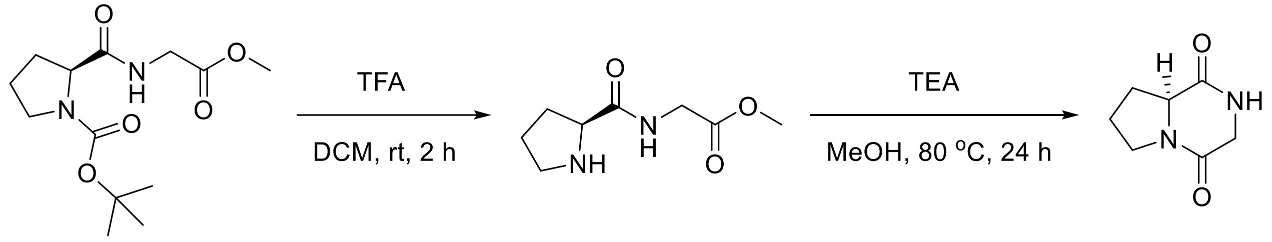


**3** **4** **5**

**Step 2**

Synthesis of (*S*)-Hexahydropyrrolo[1,2-*a*]pyrazine-1,4-dione (**5**)

To a solution of (*S*)-(-)-*N*-(*t*-Butoxycarbonyl)-prolylglycine methylester **3** (4.80 g, 16.8 mmol) in $CH_2Cl_2$ (84 mL), trifluoroacetic acid (18.7 mL, 252.0 mmol) was added and the solution was stirred at room temp for 2 hr. The solvent was removed under reduced pressure. The residue was dissolved in MeOH (84 mL) and treated with triethylamine (9.3 mL, 67.2 mmol). The reaction mixture was kept under reflux overnight. The solvent was removed under reduced pressure and the oily residue was redissolved in *i*-propanol to give (*S*)-hexahydropyrrolo[1,2-*a*]pyrazine-1,4-dione **5** as a white solid (2.0 g, 78%).

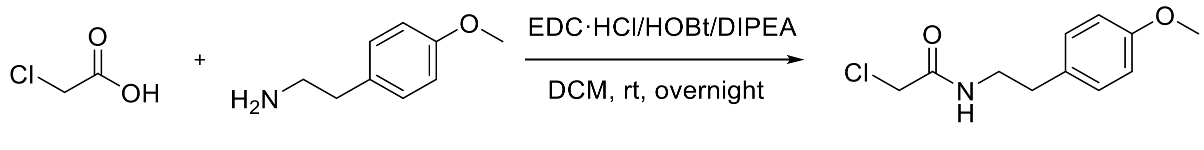


**6** **7** **8**

**Step 3**

Synthesis of 2-chloro-*N*-(4-methoxyphenethyl)acetamide (**7**)

To a solution of 2-chloroacetic acid **6** (3.00 g, 31.8 mmol) in $CH_2Cl_2$ (160 mL), 2-(4-methoxyphenyl)ethylamine **7** (4.6 mL, 31.8 mmol) and *N*,*N*-diisopropylethylamine (8.45 mL, 63.5 mmol) were added and the reaction mixture was stirred for 15 min at room temperature. After addition of 1-[3-(dimethylamino)propyl)]-3-ethylcarbodiimide hydrochloride (7.30 g, 38.1 mmol) and 1-hydroxybenzotriazole monohydrate (5.14 g, 38.1 mmol), the reaction mixture was stirred for 24 h at room temp. The reaction mixture was washed successively with 1 N HCl aqueous solution (160 mL) and sat. $NaHCO_3$ aqueous solution (160 mL). The organic layer was dried over $MgSO_4$, filtered, and concentrated. The residue was washed with diethyl ether to give 2-chloro-*N*-(4-methoxyphenethyl)acetamide **8** as a yellow solid (4.60 g, 63%).

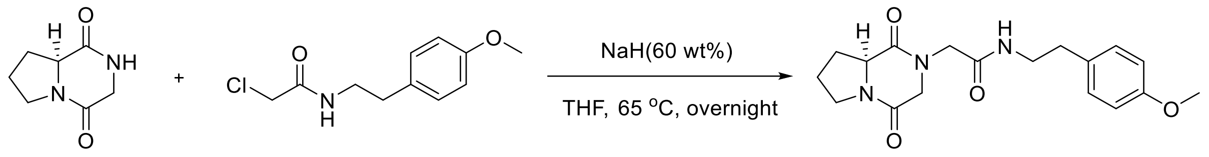


**5** **8** **9**

**Step 4**

Synthesis of (*S*)-2-(1,4-dioxohexahydropyrrolo[1,2-*a*]pyrazin-2(*1H*)-yl)-*N*-(4-methoxyphenethyl)acetamide (**9**)

To a solution of (*S*)-hexahydropyrrolo[1,2-*a*]pyrazine-1,4-dione **5** (1.00 g, 6.40 mmol) in THF (32 mL), portionwise 60 wt% NaH (260 mg, 6.40 mmol) at 0°C was added and the reaction mixture was stirred 15 min at room temperature. 2-chloro-*N*-(4-methoxyphenethyl)acetamide **7** was added slowly to the reaction mixture then the mixture was stirred overnight at 65°C. The reaction was monitored by TLC. The reaction mixture was quenched with sat. $NH_4Cl$ aqueous solution (100 mL) and extracted with EtOAc (2 X 100 mL). Combined organic layers were dried over MgSO4, filtered, and concentrated. The crude product was purified by silica-gel column chromatography (eluent: MeOH 10% in $CH_2Cl_2$) to give (*S*)-2-(1,4-dioxohexahydropyrrolo[1,2-*a*]pyrazin-2(*1H*)-yl)-*N*-(4-methoxyphenethyl)acetamide **9** as a yellow foam (1.74 g, 79%).

**Supplementary Fig. 6**
